# Carbon footprint awareness scale (CFAS): validity and reliability study on university students

**DOI:** 10.1038/s41598-025-07947-x

**Published:** 2025-08-09

**Authors:** Aydın Pekel, Keziban Yoka, Mehmet Behzat Turan, Osman Yoka, Oktay Akyüz, Osman Pepe

**Affiliations:** 1 Faculty of Sports Sciences, Marmara University, İstanbul, Turkey; 2 Vocational School of Physical Education and Sports, Istanbul Esenyurt University, İstanbul, Turkey; 3 Faculty of Sports Sciences, Erciyes University, Kayseri, Turkey; 4 Institute of Health Sciences, Erciyes University, Kayseri, Turkey; 5 Institute Of Health Sciences, Kocaeli University, Izmit, Turkey; 6 Faculty of Sports Sciences, Süleyman Demirel University, Isparta, Turkey

**Keywords:** Carbon emission, Carbon footprint, University student, Scale, Validity, And reliability, Environmental sciences, Environmental social sciences

## Abstract

This study was designed with a mixed model to develop a valid and reliable measurement tool to measure university students’ carbon footprint awareness. A total of 1053 university students, 454 female and 599 male, were included in the study voluntarily. To provide evidence for the scale’s validity, exploratory factor analysis (EFA) and confirmatory factor analysis (CFA) were applied to the measurement tool within the scope of construct validity. In the Exploratory Factor Analysis (EFA) conducted in the first stage of the study, and the Confirmatory Factor Analysis (CFA) in the second stage, scale questions consisting of 50 items (*n* = 500) were applied to the participants. The higher the variance from exploratory factor analysis, the stronger the scale’s factor structure. Our item loadings vary between 0.40 and 0.70. These ratios show that the items in our scale are significant. No item was removed from the scale due to the item analysis. One modification process was made. It was proven that the Cronbach’s Alpha coefficient, tested in line with the reliability of the measurement tool, was high. As a result of the analyzes performed, Cronbach’s Alpha reliability coefficients; For “Buildings factor” it was found as 0.783, for “Fossil Fuel Transportation and Transportation factor” it was found as 0.873, for “Oil Refinery factor” it was found as 0.857, for “Industry factor” it was found as 0.826, for “Electricity and Heat Production factor” it was found as 0.807, for “Land Use and Forestry factor” it was found as 0.790, for “Agricultural Activity factor” it was found as 0.858, for “Waste factor” it was found as 0.781, for “Other Energy Production factor” it was found as 0.864, for “Sports Organizations factor” it was found as 0.910 and for “Carbon Footprint Awareness Scale” it was found as 0.966. As a result of confirmatory factor analysis, the χ2/sd value, one of the fit indexes of the model, was determined as 2.79. Accordingly, it was determined that the χ2/sd ratio was the perfect fit for the analysis. In the second stage of the study, scale questions consisting of 50 items (*n* = 553) were applied to the participants. In the CFA applied to the obtained data, the value of χ2/sd, one of the fit indexes of the model, was determined as 2.75. In CFA, the Chi-Square/degrees of freedom (df) ratio being below three corresponds to a perfect fit, and below five corresponds to a moderate fit. In this direction, it was determined that the χ2/sd ratio was perfect for the analysis. RMSEA value showed a good fit at a 0.056 level. Thus, it was determined that the 50-item and 10-factor structure of the scale was confirmed as a model. In this context, it can be said that the Carbon Footprint Awareness Scale is a valid and reliable data collection tool that can measure carbon footprint awareness to create awareness and a conscious society among university students and the society in general.

## Introduction

Humans have started to affect and be affected by nature since the moment they came into being. They wanted to benefit from nature’s resources for good living conditions and entered a period where consumption reached much higher than production^1,2^. The demand for food, energy, and production-based sectors is increasing every passing day in our world, and this situation increases the establishment of higher capacity production systems and the orientation towards integrated production activities. In recent years, especially thanks to the increase in the current incentives in the renewable energy sector^3^ and the development of interest in renewable energy all over the world, waste and harmful substances resulting from energy production have started to decrease, and an awareness has been created on this issue. Similarly, industry-based production systems generally create side products and wastes that can be categorized and measured. However, the waste from national and international sports activities and the harmful wastes they create have been neglected and not monitored for a long time because they are more difficult to measure. At this point, the carbon footprint allows the measurement of harmful wastes resulting from sports activities, the determination of the amount of waste they create compared to other sectors, and the implementation of actions to prevent this^4^. The concept of carbon footprint is mainly measured in carbon dioxide equivalent units and is an important indicator of the environmental consequences of human actions^5,6^. The first step in reducing the effects of human-induced activities on natural systems is awareness: Accepting the existence of environmental problems, clarifying the sources of the problems, and searching for and implementing solutions in this direction. In order to increase the awareness mentioned and create behavioral change, social consciousness should be increased^7^. At this point, it is important to benefit from the theories used to understand and examine human behavior in social psychology. The most important of these theories is the Planned Behavior Theory, which is used to analyze human behavior. Theory of Planned Behavior is a model used to determine intentions to predict and understand human behavior^8^. The theory of planned behavior states that intention is the primary explanatory factor for individuals to perform a behavior; attitudes explain individuals’ intentions, perceived social pressure (subjective norms), and perceived behavioral control. In addition, this theory focuses on the specific consumer behavior of interest rather than the general evaluation or benefits of a product or service^8,9^. Ajzen and Fishbein developed the theory of planned behavior. Previously, this theory was called the “Theory of Reasoned Action”; later, it was called the “Theory of Planned Behavior” and was used in various fields^10^. The current study was designed within the scope of this theory. When evaluating the carbon footprint, the direct and indirect emissions that may occur before the production phase should be calculated to cover all stages within the life cycle framework^11^. Global calculations show that the carbon footprint constitutes more than 60% of the total ecological footprint^12,13^. The carbon footprint is encountered at every stage of social life. For example, the carbon footprint is the use of airplanes by athletes and fans in national and international sports organizations, or the carbon dioxide emissions generated during the production, processing, and retail sales of jerseys, flags, and pennants purchased by fans. Therefore, in order to determine the main emission sources in the supply chains and to ensure that measures are taken to reduce emissions by informing the relevant stakeholders, to increase awareness among young individuals and to disseminate carbon footprint prevention efforts and to determine the dynamics that can be used in this dissemination movement, it is of great importance to develop a data collection tool that can measure carbon footprint in all its dimensions^14,15^. In this context, the research aims to develop a data collection tool that validly and reliably measures university students’ carbon footprint awareness. Developing a carbon footprint awareness scale (CFAS) is important because it helps individuals understand their lifestyle choices’ direct and indirect environmental impact. Explaining and validating the Carbon Footprint Awareness Scale is important for several reasons, as it can help address the basic environmental, social, and psychological aspects of climate change mitigation. A validated scale allows individuals to assess their carbon footprint, improving their awareness of how personal actions contribute to climate change^16^. Furthermore, with a validated scale, climate action programs can tailor interventions to specific individuals or groups based on their carbon footprints, making efforts more efficient and effective. By measuring their carbon footprint, people can learn how their daily activities, such as energy consumption, transportation, and waste, impact climate change^17^. Furthermore, a CFAS educates people about various sources of carbon emissions, not only from industrial activities but also from daily life. This helps build a more environmentally and nature-conscious, climate-conscious society^18^. Many studies on measuring carbon footprint are in the relevant literature^16,19–29^. The most important difference between the current study and the studies in the relevant literature is that it is a scale development study specific to university students. This study examines university students’ behavioral styles, individual awareness, and social consciousness, and evaluates their awareness on the subject on a scale. For this purpose, is the Carbon Footprint Awareness Scale (CFAS) a valid and reliable measurement tool for university students? It will be examined. As a result, by motivating individuals to take responsibility for their environmental footprint, the CFAS encourages a collective commitment to climate action and the transition to a more sustainable future^30^. At the same time, this innovative tool supports rigorous scientific research on carbon footprint awareness and encourages individuals to play an active role in global efforts to reduce climate change. By promoting awareness, informing decision-making, and encouraging sustainable behaviors, the CAF contributes to building resilience and sustainability in communities worldwide, ensuring a healthier planet for current and future generations.

## Materials and methods

### Research model

This study used a mixed model to develop and validate a valid and reliable measurement tool to measure and evaluate individuals’ carbon footprint awareness. Mixed studies are studies in which qualitative and quantitative data are considered in a single study and different data sources are transformed and verified^31–34^. This design usually starts with using qualitative data collection tools and ends with supporting the data collected with quantitative data. In a mixed research design, researchers aim to discover a concept or phenomenon through qualitative means, develop a measurement tool about that concept or phenomenon, and then test the tool^35^. The themes obtained through the qualitative data collection process are used to develop a quantitative measurement tool^36^. In the current research, priority was given to the qualitative data collection process within the framework of the mixed research design. Then, the research continued with the quantitative data collection process. It is seen that similar research designs are used in scale development studies.

### Study group (population-sample)

The study sample consists of university students over 18. Criterion sampling, a frequently used purposeful sampling method that provides in-depth research opportunities, was used in the study. In criterion sampling, the researcher can create a criterion that meets a predetermined criterion^34,37,38^.

These criteria are;

- Turkish university students,
- Studying in a formal education program at one of the universities in Turkey,

According to the criteria we determined for the study, the research population consists of 3,150,000 university students out of 7,000,000 in Turkey^39^. While determining the sample group, face-to-face interviews were conducted with 100 university students. Information about the scale to be developed was collected. Surveys were then sent to all universities using an online platform. Our sample group consists of 1053 people who volunteered to participate in our study and completed our survey. When the research population is taken into consideration, when the table where the sample sizes to be taken from various population sizes for sampling errors of 0.01, 0.02, 0.03, 0.04, and 0.05 are calculated, it was seen that the number of samples is sufficient^40^. The research was conducted in two stages. In the first stage (pilot study), a total of 500 university students participated, 207 female and 293 male. In the second stage, 553 people participated, including 247 female and 306 male university students. There are opinions in the literature that the study group should be five times the number of items in the developed scale^33,41–45^. Therefore, it can be said that the working group size for the two stages is reasonably sufficient. The data of the study were collected between 02.01.2025 and 04.02.2025.

### Creating the scale item pool

This process progressed in five stages. In the first stage, literature on “carbon footprint awareness” and “carbon footprint sub-dimensions” to be used in the scale was reviewed.

In scale development studies, first of all, the theoretical structure of the feature to be measured and the sub-dimensions of the feature in line with the theory must be investigated^46^. In the second stage, after the literature review, face-to-face interviews (qualitative interviews) were conducted with 100 (50 female, 50 male) university students who volunteered to participate in the research.

The 100 students who participated in the face-to-face interviews were not included in the sample of 1053 participants used in the study’s quantitative stages (EFA and CFA). These interviews were conducted before the item pool development and served as a qualitative pre-study to understand university students’ perceptions, awareness, and experiences related to carbon footprint. The interviews aimed to gather rich, real-world insights to inform item generation and ensure that the language and themes used in the scale reflected the lived experiences of the target population.

The interview questions focused on:

- Students’ understanding of the term “carbon footprint”.
- Daily activities that they believe contribute to carbon emissions.
- Their awareness of personal and societal responsibilities related to climate change.
- Their attitudes toward environmental sustainability.

The results of these interviews were thematically analyzed and directly contributed to the generation of the initial 146-item pool by identifying key themes and behavior patterns, which were later refined through expert review and item analysis. These qualitative findings ensured content validity and contextual relevance of the items.

In the third stage, relevant studies on carbon footprint^16,47–49^. They were used in the article writing process. According to Tavşancıl^42^, since the number of items written should be around 100, care was taken to ensure that the number of items was written at a sufficient level during the preparation process of the item pool. Since it was thought that insufficient, unclear, incomprehensible, and unrepresentative items would be removed, the items created for the item pool were kept as many as possible. In this context, the first item pool created included 146 items. The item pool was presented to expert opinion by creating appropriate, inappropriate, and correctable boxes next to each item in the item pool.

In the fourth stage, six expert opinions were consulted, including two environmental engineers with a doctorate, one measurement and evaluation specialist, one civil engineer, one sports scientist, and 1 Turkish language and literature specialist for Turkish language support. The purpose of obtaining expert opinion is to ensure scope validity. Content validity determines whether each item in the scale suits the desired awareness feature through “expert opinion”^34,50^. For content validity, each expert examined the items in the item pool regarding clarity of expression, plain and appropriate language, and meeting the specification. The reduction from 146 to 83 items was based on expert evaluations in environmental science, measurement and evaluation, and educational psychology. A total of six experts reviewed the items in terms of content relevance, clarity, and representativeness of the construct.

The experts were asked to rate each item using a 4-point scale (1 = not relevant, 4 = highly relevant). Items with a Content Validity Ratio (CVR) below the acceptable threshold (based on Lawshe’s table) were excluded or revised. As a result of this content validity process, 63 items were removed due to redundancy, low relevance, or ambiguous wording.

A 7-point Likert-type scale (Strongly Disagree, Disagree, Somewhat Disagree. Neither Disagree nor Disagree, Somewhat Agree, Agree, Strongly Agree) was used, which prevents participants from reaching extreme points in their answers and allows for balanced selection^51^, increases the validity and reliability of the scale by ensuring that the answers are expressed sensitively^52^, and provides healthier results in statistical analyses due to the wide distribution^53^. Participants were asked to evaluate their carbon footprint in ten categories.

**Fig. 1:**
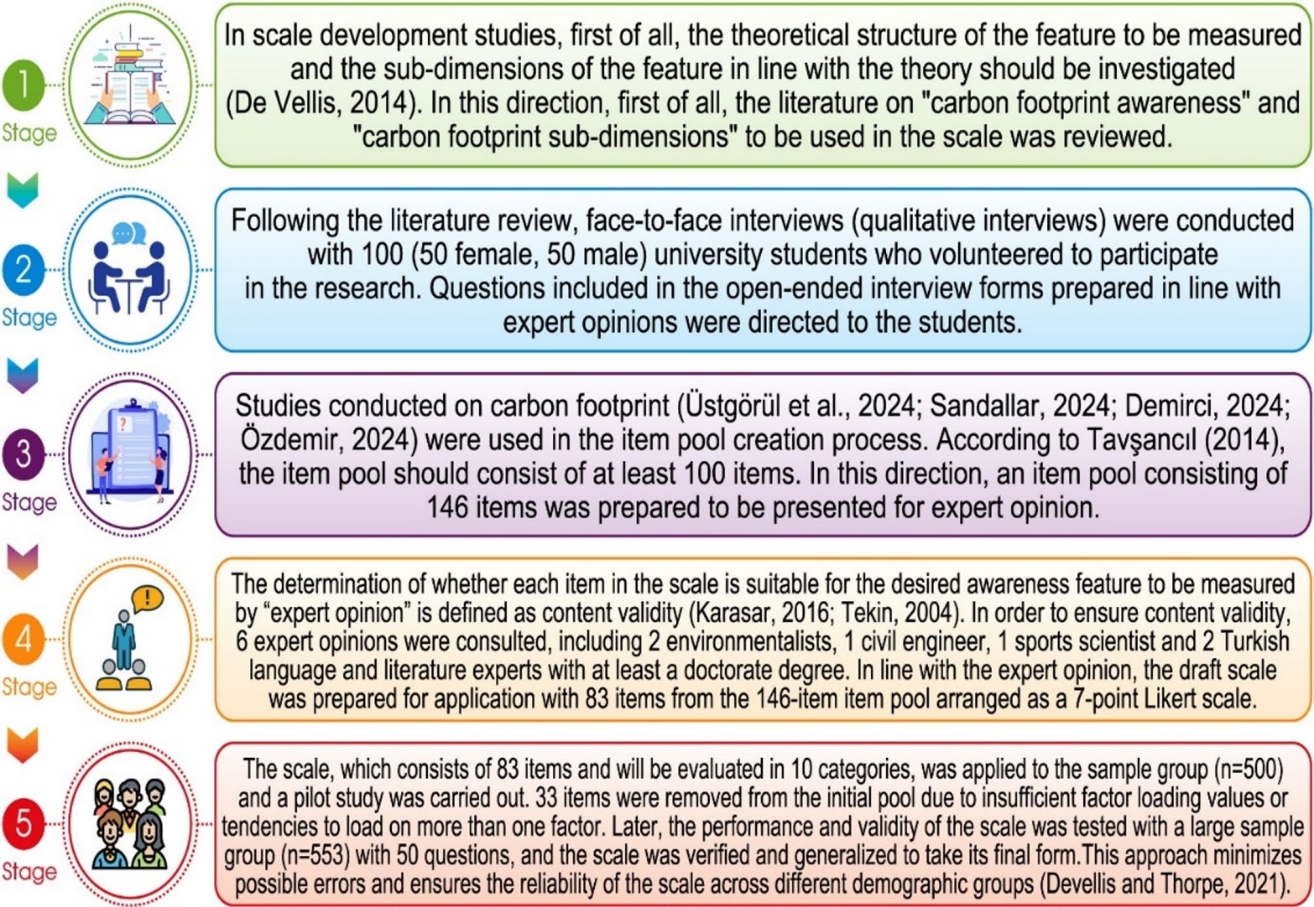
Flow Chart for Scale development procedure.

A pilot study was implemented in the fifth stage with our sample group (*n* = 500) and 83 questions. In pilot studies, questions are tested with a sample group to determine content errors and the level of comprehensibility. To ensure the accuracy and interpretability of the factors, items with factor loading values below the recommended threshold of 0.30 were systematically removed from the scale. Additionally, items showing cross-loading on more than one factor were excluded to maintain the integrity of the scale and align with methodological recommendations^54,55^. Thirty-three items were removed from the initial pool due to insufficient factor loading values or tendencies to load on more than one factor. Later, the performance and validity of the scale were re-applied to a large sample with 50 questions (*n* = 553), the final version of the scale was verified, and its generalizability was tested. This approach helps minimize potential errors and ensure the scale’s reliability across the different demographic groups^56,57^. The research procedure is given in Fig. 1 below.

### Analysis of data

In order to determine the tests to be used for the analysis of the study, the Kolmogorov-Smirnov test was performed on the data, and the skewness-kurtosis values were examined. George and Mallery^58^ consider the ± 2 range within acceptable limits for the normality assumption. When the kurtosis and skewness values in the study are examined, it is seen that they are within these limits. Descriptive statistics and frequency values are given. At the same time, Pearson correlation values and Fisher z-score values, which measure the strength and direction of the linear relationship between two continuous variables, were also examined. Exploratory Factor Analysis (EFA) and Confirmatory Factor Analysis (CFA) were conducted to provide evidence for the scale’s construct validity. In this process, the “varimax” rotation technique was also examined to identify more meaningful and easy factors; item test correlations were examined to prove the item analyses; and the Kaiser-Meyer Olkin (KMO) coefficient and Bartlett Sphericity test results were examined to determine the suitability of the data for the principal component analysis. One of the important steps in scale development is ensuring reliability. Therefore, test-retest and Crα α reliability coefficient values were examined. In order to provide evidence for validity and reliability, it was analyzed using SPSS 26 and LISREL 8.80 programs, and item analyses were evaluated.

## Result

When we examine the socio-demographic characteristics of the university students who participated in the research in the pilot study, a total of 500 volunteer students, 207 female and 293 male, participated. 80.2% of the participants are between the ages of 18–22, and 19.8% are between the ages of 23–27. 16% of the students stated that they are in the first grade, 27.2% in the second grade, 30% in the third grade, and 26.8% in the fourth grade. When we examine the socio-demographic characteristics of the university students who participated in the study, 247 females and 306 males, 553 volunteer students participated. 77.8% of the participants are between the ages of 18–22, and 22.2% are between the ages of 23–27. 18.1% of the students stated that they are in the 1st grade, 26.6% are in the 2nd grade, 30.4% are in the 3rd grade, and 25% are in the 4th grade (Table 1).

**Table 1:** Descriptive statistics of participants, frequency values.

Perform	Variable	Category	F	%
Pilot study	Grade	Woman	207	41.4
		Man	293	58.6
	Age	18–22 age	401	80.2
		23–27 age	99	19.8
	Class	1. Grade	80	16.0
		2. Grade	136	27.2
		3. Grade	150	30.0
		4. Grade	134	26.8
Scale carry out	Gender	Woman	247	44.7
		Man	306	55.3
	Age	18–22 age	430	77.8
		23–27 age	123	22.2
	Grade	1. Grade	100	18.1
		2. Grade	147	26.6
		3. Grade	168	30.4
		4. Grade	138	25.0

F: Frequency %: Percent.

**Table 2:** Results of KMO.

KMO and Bartlett’s Test		
Kaiser-Meyer-Olkin measure of sampling adequacy		0.948
Bartlett’s test of sphericity	Approx. Chi-Square	15474.121
	Df	1431
	Sig.	0.000

df = degrees of freedom; Sig. = significance (p-value).

In order to determine the suitability of the data for principal component analysis, the Kaiser-Meyer Olkin (KMO) coefficient and Bartlett’s Sphericity test results were examined. One of the important steps in scale development is to ensure reliability. The Kaiser value was determined as 0.948 from Bartlett and KMO measurements. The result shows the sample size adequacy and the data set’s suitability^59,60^ (Table 2).

**Table 3:** Total variance explained.

Total variance explained									
Component	Initial Eigenvalues			Extraction sums of squared loadings			Rotation sums of squared loadings		
	Total	% of Variance	Cumulative %	Total	% of Variance	Cumulative %	Total	% of Variance	Cumulative %
1	19.281	35.706	35.706	19.281	35.706	35.706	5.515	10.212	10.212
2	3.220	5.962	41.668	3.220	5.962	41.668	4.200	7.778	17.990
3	2.019	3.739	45.407	2.019	3.739	45.407	4.114	7.618	25.609
4	1.645	3.047	48.454	1.645	3.047	48.454	3.678	6.811	32.419
5	1.514	2.804	51.259	1.514	2.804	51.259	3.434	6.358	38.778
6	1.333	2.468	53.727	1.333	2.468	53.727	2.977	5.514	44.291
7	1.283	2.376	56.103	1.283	2.376	56.103	2.877	5.328	49.619
8	1.218	2.255	58.357	1.218	2.255	58.357	2.863	5.302	54.921
9	1.099	2.034	60.392	1.099	2.034	60.392	2.089	3.868	58.789
10	1.001	1.854	62.245	1.001	1.854	62.245	1.866	3.456	62.245

Extraction Method: Principal Component Analysis.

**Fig. 2:**
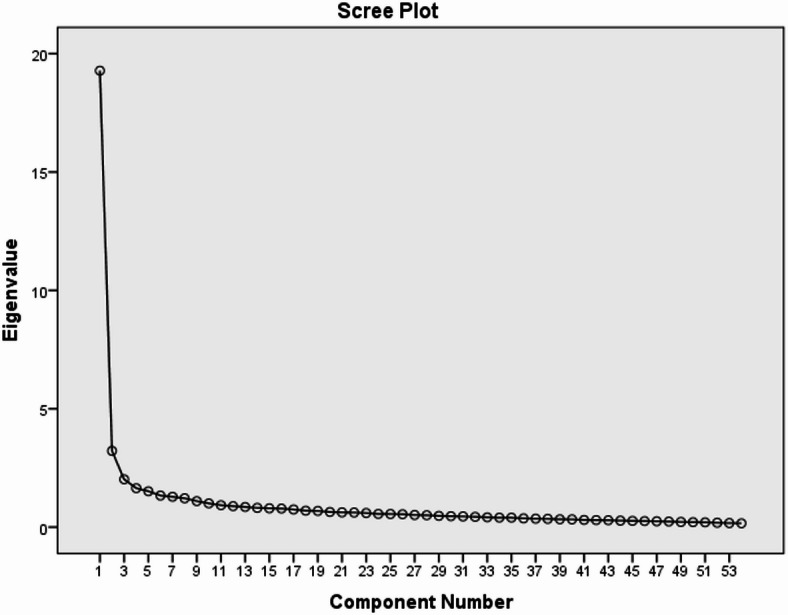
Scatter plot of explanatory factor analysis.

Table 3 shows the eigenvalues of the items related to the carbon footprint awareness scale. The eigenvalues were found to be ten factors greater than 1. The first factor explains 10% of the variance, the second factor explains 18% of the variance, the third factor explains 26% of the variance, the fourth factor explains 32% of the variance, the fifth factor explains 39% of the variance, the sixth factor explains 44% of the variance, the seventh factor explains 50% of the variance, the eighth factor explains 55% of the variance, the ninth factor explains 59% of the variance, and the tenth factor explains 62% of the variance. Factor item distributions are given in Fig. 2.

**Table 4:** Carbon footprint awareness exploratory factor analysis.

No	Scale items	Components									
		1	2	3	4	5	6	7	8	9	10
1	The energy used to produce building materials increases the amount of carbon emissions.	0.681									
2	The electricity used to run construction machinery increases carbon emissions.	0.649									
3	Reducing residential electricity use reduces carbon emissions.	0.674									
4	The energy used to transform construction waste generated during the demolition of buildings increases the amount of carbon emissions.	0.564									
5	The use of lighting equipment in residences increases carbon emissions.	0.516									
6	Extracting fossil fuels increases carbon emissions.		0.579								
7	Methane leaks occurring during natural gas transportation increase the amount of carbon.		0.508								
8	Processing fossil fuels increases carbon emissions.		0.688								
9	Fossil fuels used in airplanes increase the amount of carbon emissions.		0.599								
10	Fossil fuels used in rail transportation increase the amount of carbon emissions.		0.649								
11	Fossil fuels used in maritime transportation increase the amount of carbon emissions.		0.646								
12	Fuel: One of the vehicles used in freight transport increases the amount of carbon emissions.		0.463								
13	The large amount of energy consumed while processing crude oil increases carbon emissions.			0.460							
14	Wastewater generated during sewage processing increases carbon emissions.			0.453							
15	When gasoline from a refinery is burned, it produces carbon emissions.			0.649							
16	When diesel from the refinery is being processed, emissions are generated.			0.707							
17	The use of fossil fuels for industrial production increases the amount of carbon emissions.				0.536						
18	Raw materials used in cosmetic production increase the amount of carbon emissions.				0.540						
19	Carbon emissions increase during the removal or recycling of waste generated in production processes.				0.650						
20	Raw materials used in textile production increase the amount of carbon emissions.				0.673						
21	Cement factories increase the amount of carbon emissions.				0.652						
22	Choosing energy-efficient lighting devices reduces the amount of carbon emissions.					0.439					
23	Energy production with renewable energy sources reduces the amount of carbon emissions.					0.617					
24	Items used to save energy reduce the amount of carbon emissions.					0.623					
25	Forests reduce the amount of carbon emissions.						0.712				
26	Forest fires increase carbon emissions.						0.649				
27	Afforestation reduces the amount of carbon emissions in the air.						0.725				
28	Pesticides used in agricultural lands increase carbon emissions.						0.433				
29	Agricultural activities increase carbon emissions.							0.714			
30	The amount of carbon emissions increases during the food production stages.							0.589			
31	Livestock activities increase the amount of carbon emissions.							0.665			
32	Reducing the consumption of animal products reduces the amount of carbon emissions.							0.630			
33	The use of commercial fertilizers increases the amount of carbon emissions.							0.591			
34	The methods used to produce animal products increase the amount of carbon emissions.							0.637			
35	The use of organic fertilizers increases the amount of carbon emissions.							0.722			
36	Buying recycled, eco-friendly products reduces the amount of carbon emissions.								0.652		
37	Scrap metal recycling reduces carbon emissions.								0.514		
38	The reason for the increase in carbon emissions is the waste treatment systems.								0.748		
39	Wastewater treatment processes increase the amount of carbon emissions.								0.722		
40	Fossil fuels used in energy production increase the amount of carbon emissions.									0.570	
41	The energy used for nuclear power plant construction increases the amount of carbon emissions.									0.716	
42	Wells opened for energy extracted from underground resources also increase the amount of carbon emissions used in energy.									0.606	
43	The energy used for uranium enrichment increases the amount of carbon emissions.									0.586	
44	The energy used to light sports facilities increases the amount of carbon emissions.										0.641
45	The materials used in the construction of sports facilities increase the amount of carbon emissions.										0.671
46	Travel to participate in sports events increases the amount of carbon emissions.										0.701
47	The energy used during the transportation of food consumed in sports organizations increases the amount of carbon emissions.										0.659
48	The energy used to heat sports facilities increases the amount of carbon emissions.										0.623
49	The energy used to cool sports facilities increases the amount of carbon emissions.										0.707
50	The energy used in the production phase of sportswear products increases the amount of carbon emissions.										0.670

Extraction Method: Principal Component Analysis. Rotation Method: Varimax with Kaiser Normalization. a. Rotation converged in 8 iterations. There are no reverse-scored items in our scale.

Example item: “The use of fossil fuels for industrial production increases the amount of carbon emissions.” (7-point Likert scale: 1 = Strongly Disagree, 7 = Strongly Agree).

When Table 4 is examined;

1. **Building**: This factor covers items 1, 2, 3, 4, and 5 of the scale and focuses on determining the energy used by individuals in buildings and the carbon emissions that occur during the construction of buildings.
2. **Fossil Fuel Transportation and Transportation**: This factor covers items 6, 7, 8, 9, 10, 11, and 12 of the scale and focuses on determining the carbon emissions that occur during the transportation of coal, natural gas, and oil and are related to the transportation preferences of individuals.
3. **Petroleum Refining**: This factor covers items 13, 14, 15, and 16 of the scale and focuses on the carbon emissions that occur while extracting oil from the ground and converting it into useful gases.
4. **Industry**: This factor covers items 17, 18, 19, 20, and 21 of the scale and focuses on carbon emissions during production activities and raw material processing or transportation.
5. **Electricity and Heat Production**: This factor covers items 22, 23, and 24 of the scale and focuses on the carbon emissions that occur due to the energy used by individuals for lighting and heating.
6. **Land Use and Forestry**: This factor covers items 25, 26, 27, and 28 of the scale and focuses on the impacts individuals have on nature as part of their production and consumption activities.
7. **Agricultural Activities**: This factor covers items 29, 30, 31, 32, 33, 34, and 35 of the scale and focuses on carbon emissions from substances such as medicines, fertilizers, etc., used during agricultural activities.
8. **Waste**: This factor covers items 36, 37, 38, and 39 of the scale and focuses on individuals’ practices and awareness regarding waste production, disposal methods, and recycling efforts.
9. **Other Energy**: This factor covers items 40, 41, 42, and 43 of the scale and focuses on carbon emissions from energy activities used in production, distribution, and transportation.
10. **Sports organizations**: This factor covers items 44, 45, 46, 47, 48, 49, and 50 of the scale and focuses on carbon emissions occurring in national and international sports organizations.

**Table 5:** Factor names, sample items and reliability.

	Factor name	Number of Items	Cronbach’s α	Skewness	Kurtosis
1	Building	5	0.783	− 0.466	0.381
2	Fossil fuel transportation	7	0.873	− 0.666	0.647
3	Oil refining	4	0.857	− 0.729	0.599
4	Industry	5	0.826	− 0.582	0.330
5	Electricity and heat generation	3	0.807	− 0.794	0.616
6	Land use and forestry	4	0.790	− 0.777	0.292
7	Agricultural activities	7	0.858	− 0.448	0.049
8	Waste	4	0.781	− 0.612	0.593
9	Other energy production	4	0.864	− 0.830	0.948
10	Sports organizations	7	0.910	− 0.586	0.273
Total	Carbon footprint awareness scale	50	0.966	− 0.260	0.295

**Fig. 3:**
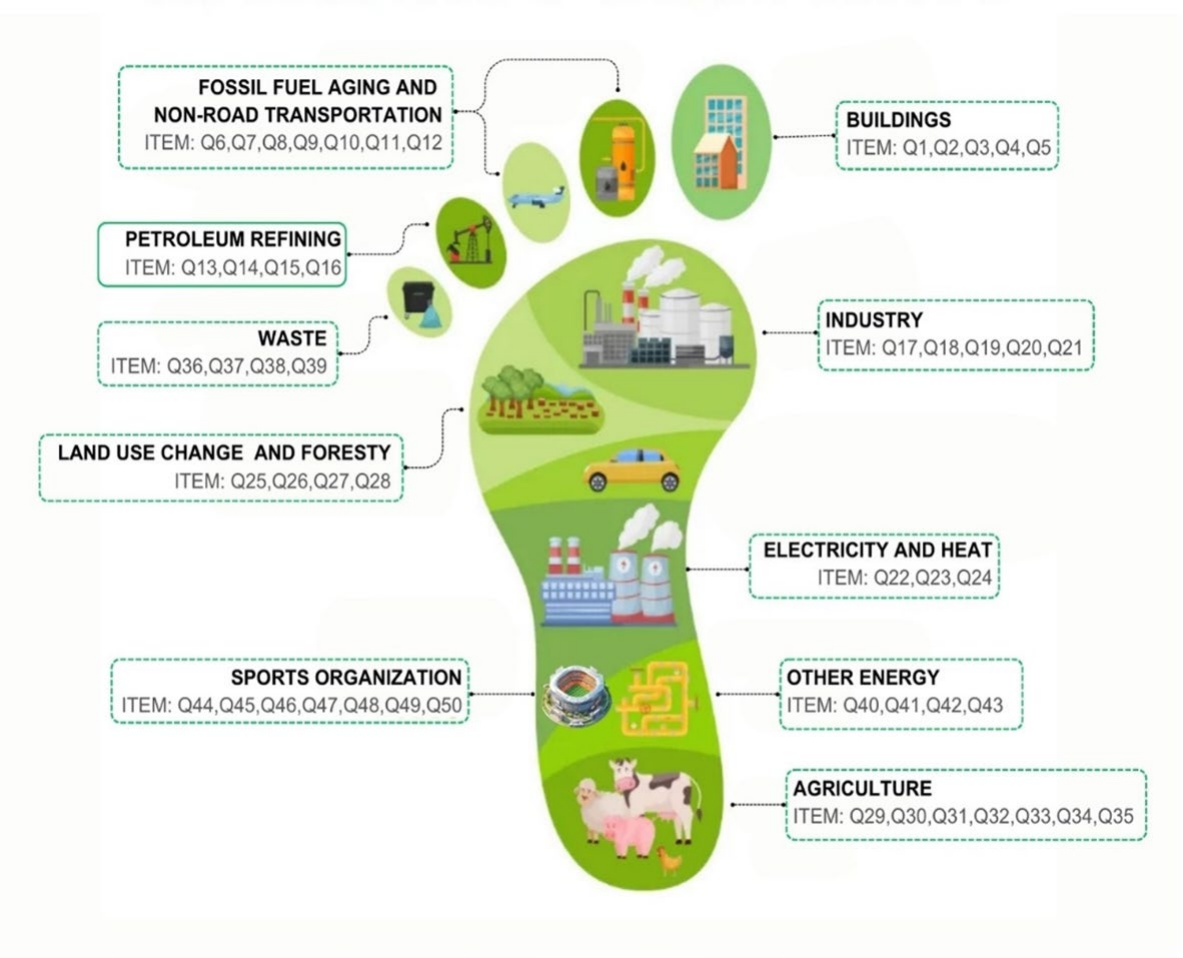
Distribution of scale questions.

When Table 5 is examined, regarding the reliability of the scale, Cronbach’s Alpha reliability coefficients were calculated for the entire scale and each sub-dimension separately. Since the Cronbach Alpha coefficient is calculated by taking into account all questions and consistent statistical bases, it is the coefficient that best reflects the general reliability structure of the test compared to other coefficients^57^. According to experts, if the alpha coefficient is between 0.70 and 1, the scale has high reliability^33,42,57,61,62^. As a result of the analyzes, Cronbach’s Alpha reliability coefficients were determined as 0.783 for the “Buildings factor”, 0.873 for the “Fossil Fuel Transportation and Transportation factor”, 0.857 for the “Petroleum refining factor”, 0.826 for the “Industry factor”, 0.807 for the “electricity and heat production factor”, 0.790 for the “land use and forestry factor”, 0.858 for the “agricultural activity factor”, 0.781 for the “waste factor”, 0.864 for the “other energy production factor”, 0.910 for the “sports organizations factor” and 0.966 for the “carbon footprint awareness scale”. The distribution of the scale’s sub-dimensions is presented in Fig. 3.

Researchers have made different suggestions about which fit indices should be reported. McDonald and Ho^63^; CFI, GFI, NFI, and NNFI (TLI); Garver and Mentzer^64^; RMSEA, CFI, and NNFI (TLI); Brown^65^; RMSEA, SRMR, CFI, and NNFI (TLI); Iacobucci^66^ recommends reporting CFI and SRMR fit indices. Gerbing and Anderson^67^ state that different fit indices can be reported depending on the researcher’s aim. The NFI value of the fit indices was determined as 0.96; NNFI (TLI) value as 0.97; GFI value as 0.91; CFI value as 0.97; AGFI value as 0.86; SRMR as 0.37; PNFI as 0.96, and PGFI as 0.98. An RMSEA value less than 0.05 indicates excellent fit, and an RMSEA value less than 0.08 indicates good fit^33,68^. It was determined that a good fit was provided with an RMSEA value of 0.060. The RMSEA value shows an acceptable fit with a level of 0.060. Thus, it was determined that the 50-item and 10-factor structure of the scale was confirmed as a model. When compared with the standard fit criteria that should be considered as a result of the confirmatory factor analysis stated in the study of Schermelleh-Engel and Moosbrugger^69^;, it is seen that the overall fit values are in the group of “best and acceptable fit values” and the RMSEA value is within an acceptable value. In the second stage of the study, scale questions consisting of 50 items (*n* = 500) were applied to the participants. In the CFA applied to the obtained data, the value of χ2/sd, one of the fit indices of the model, was determined as 2.97. A Chi-Square/degree of freedom ratio (df) in the CFA below 3 corresponds to excellent fit, and a value below 5 corresponds to moderate fit^70,71^. In this direction, it was determined that the χ2/sd ratio showed an excellent fit for the analysis performed. The RMSEA value showed a good fit with a level of 0.062. Since fit indices have strengths and weaknesses compared to each other in evaluating the fit between the theoretical model and real data, it is recommended to use many fit index values to reveal the model’s fit. In the most commonly used fit indexes, the NFI value was determined as 0.89; the NNFI value as 0.93; the GFI value as 0.81; the CFI value as 0.90; and the AGFI value as 0.85. It is understood that the fit indexes are between good and acceptable fit values. At this analysis stage, it was decided that the modification suggestions should be evaluated. The error variances of some items were associated to obtain a decrease in the Chi-square value (improve the model). When we look at the modification suggestions of the LISREL program, the association suggestions between various items were evaluated. The suggestion for the most significant decrease in the Chi-square value will be obtained from the association of items A7 and A8. However, it is necessary to have a theoretical or solid logical basis when making the association^68^. When we look at items A7-A8, it is seen that both items are under the same dimension and have semantically similar expressions. One modification process was applied. The most commonly used fit indices were determined as NFI value 0.89, NNFI (TLI) value 0.93, GFI value 0.81, CFI value 0.90, AGFI value 0.85, SRMR 0.34, PNFI 0.95, and PGFI 0.94 (Table 6).

**Table 6:** Fit values ​​for the scale.

Scale related values										
NFI	NNFI (TLI)	IFI	RFI	CFI	GFI	AGFI	SRMR	PNFI	RMR	PGFI
0.96	0.97	0.97	0.96	0.97	0.81	0.86	0.057	0.96	0.56	0.98
X^2^/df = 2.79; RMSEA = 0.060										

**Fig. 4:**
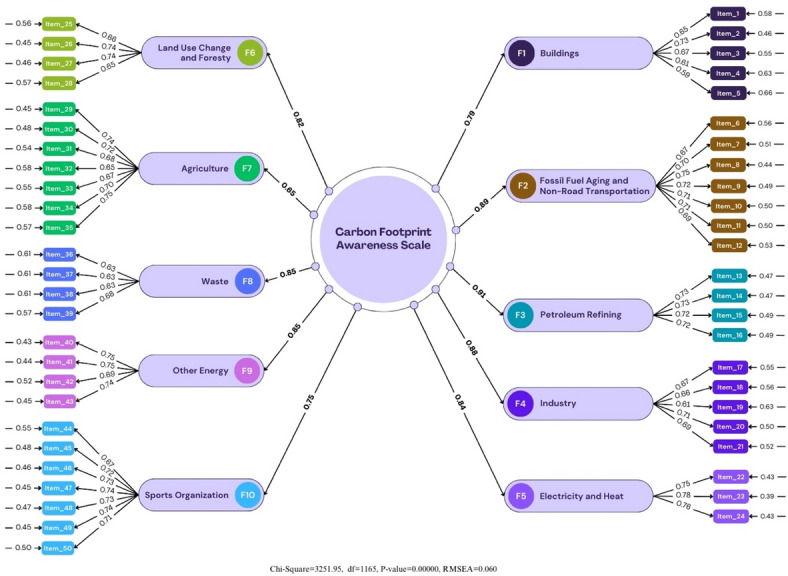
Confirmatory factor analysis results.

In the Confirmatory Factor Analysis (CFA) conducted in the first stage of the study, scale questions consisting of 50 items (*n* = 500) were applied to the participants. When Fig. 4 is examined, the value of χ2/sd, one of the fit indexes of the model, was determined as 2.79. A Chi-Square/degree of freedom ratio (df) in the CFA below 3 corresponds to the perfect fit, and a ratio below 5 corresponds to a medium-level fit^61,71^. In this direction, it was determined that the χ2/sd ratio was the perfect fit for the analysis.

**Table 7:** Fit values ​​for the scale.

Scale related values										
NFI	NNFI (TLI)	IFI	RFI	CFI	GFI	AGFI	SRMR	PNFI	RMR	PGFI
0.96	0.97	0.98	0.96	0.98	0.92	0.91	0.042	0.98	0.51	0.96
X^2^/df = 2.75; RMSEA = 0.056										

Considering the fit index recommendations of McDonald and Ho^63^. Garver and Mentzer^64^, Brown^65^, and Iacobucci^66^, the fit indexes were determined as NFI value 0.96, NNFI value 0.97, GFI value 0.92, CFI value 0.98, AGFI value 0.91, SRMR 0.42, PNFI 0.98, and PGFI 0.96. An RMSEA value less than 0.05 indicates a perfect fit, and an RMSEA value less than 0.08 indicates a good fit. A good fit was determined with an RMSEA value of 0.056. In this direction, the χ2/sd ratio for the analysis performed revealed an excellent fit. The RMSEA value showed a perfect fit with a level of 0.056. Thus, it was determined that the 50-item and 10-factor structure of the scale was confirmed as a model When compared with the standard fit criteria that should be considered as a result of the confirmatory factor analysis stated in the study of Schermelleh-Engel and Moosbrugger^69^, it is seen that the overall fit values are in the group of “perfect and acceptable fit values” (Table 7).

**Fig. 5:**
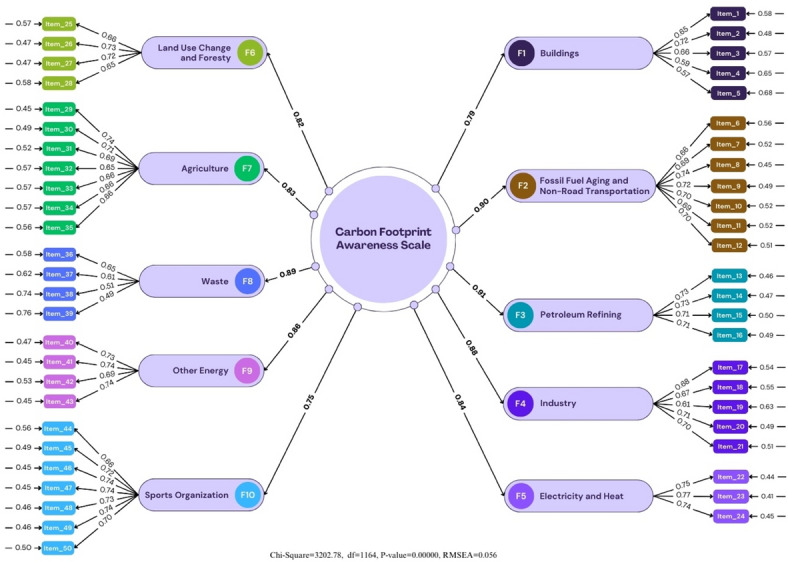
Confirmatory factor analysis results (last sample).

When Fig. 5 is examined, it is seen that there is a slight decrease in the error variances after one modification application and significant decreases in chi-square and RMSEA values. The condition that the Chi-Square/degree of freedom ratio (df) in the CFA should be below three was sought. The (χ2/sd) ratio calculated with the CFA was 2.75, and this value revealed that the proposed factor model showed a perfect fit with the data^71,72^. There are different opinions about the minimum level of item factor loadings. While it is emphasized that the item factor loading should be at least 0.40^73^, it is reported that it is a better measure if this loading value is 0.45 and above^33^. However, Hair, Black, Babin, and Anderson^74^, reported that if the item factor loading values are between 0.30 and 0.40, it meets the minimum level for interpretation, when it is 0.50 or above, it is considered meaningful in practice, and structures exceeding 0.70 are an indicator of a well-defined structure. It is stated that the item factor loadings should be 0.30 as a minimum due to the application of factor analysis. However, if the item factor loadings are between 0.30 and 0.59, it is reported that it is medium, and 0.60 and above is high^75^. The higher the variance obtained from factor analysis, the stronger the scale’s factor structure. Our item loadings vary between 0.40 and 0.70. These rates show that the items in our scale are significant.

**Table 8:** Correlation of carbon footprint scale sub-dimension scores (*n* = 553).

Sub-dimensions	*r*	1	2	3	4	5	6	7	8	9	10	11
Building ^1^	r	1										
Fossil Fuel Transportation and Transport ^2^	r	.59^**^	1									
Petroleum Refining ^3^	r	.56^**^	.75^**^	1								
Industry ^4^	r	.54^**^	.64^**^	.64^**^	1							
Electricity and Heat Production ^5^	r	.53^**^	.59^**^	.63^**^	.56^**^	1						
Land Use and Forestry ^6^	r	.44^**^	.64^**^	.56^**^	.61^**^	.57^**^	1					
Agriculture Activity ^7^	r	.46^**^	.43^**^	.42^**^	.54^**^	.47^**^	.40^**^	1				
Waste^8^	r	.54^**^	.60^**^	.55^**^	.60^**^	.48^**^	.52^**^	.51^**^	1			
Other Energy Production ^9^	r	.53^**^	.64^**^	.61^**^	.64^**^	.63^**^	.57^**^	.48^**^	.50^**^	1		
Sports Organization ^10^	r	.52^**^	.55^**^	.54^**^	.57^**^	.57^**^	.47^**^	.60^**^	.50^**^	.56^**^	1	
Carbon Footprint Awareness Scale ^11^	r	.74^**^	.83^**^	.80^**^	.81^**^	.75^**^	.74^**^	.73^**^	.74^**^	.78^**^	.80^**^	1

Note. * *p* < 0.05, ** *p* < 0.01, *N* = 1053. 1 = Buildings; 2 = Fossil fuel transportation and transport; 3 = Petroleum refining; 4 = Industry; 5 = Electricity and heat production; 6 = Land use and forestry; 7 = Agricultural activities; 8 = Waste; 9 = Other energy production; 10 = Sports organizations; 11 = Carbon footprint awareness scale.

Carbon footprint awareness: Fisher conversions of the participants participating in the study were calculated as follows. With the buildings sub-dimension, fossil fuel transportation and transportation (*r* = 0.59) resulted in a z-score of 0.67, petroleum refining (*r* = 0.56) resulted in a z-score of 0.63, industry (*r* = 0.54) resulted in a z-score of 0.60, electricity and heat production (*r* = 0.53) resulted in a z-score of 0.59, land use and forestry (*r* = 0.44) resulted in a z-score of 0.47, agricultural activities (*r* = 0.46) resulted in a z-score of 0.49, waste (*r* = 0.54) resulted in a z-score of 0.60, other energy production (*r* = 0.53) resulted in a z-score of 0.59, sports organizations (*r* = 0.52) resulted in a z-score of 0.57 and the carbon footprint awareness scale (r=, 740) resulted in a z-score of 0.95. Fossil fuel transportation and transportation sub-dimension and petroleum refining (*r* = 0.75) resulted in a z-score of 0.97, industry (*r* = 0.64) resulted in a z-score of 0.75, electricity and heat production (*r* = 0.59) resulted in a z-score of 0.67, land use and forestry (*r* = 0.64) resulted in a z-score of 0.75, agricultural activities (*r* = 0.43) resulted in a z-score of 0.45, waste (*r* = 0.60) resulted in a z-score of 0.69, other energy production (*r* = 0.64) resulted in a z-score of 0.75, sports organizations (*r* = 0.55) resulted in a z-score of 0.61, and carbon footprint awareness scale (*r* = 0.83) resulted in a z-score of 1.18. Petroleum refining and industry (*r* = 0.64) with a z-score of 0.75, electricity and heat production (*r* = 0.63) with a z-score of 0.74, land use and forestry (*r* = 0.56) with a z-score of 0.63, agricultural activities (*r* = 0.42) with a z-score of 0.44, waste (*r* = 0.55) with a z-score of 0.61, other energy production (*r* = 0.61) with a z-score of 0.70, sports organizations (*r* = 0.54) with a z-score of 0.60, carbon footprint awareness scale (*r* = 0.80) resulted in a z-score of 1.09. Industry, electricity, and heat production (*r* = 0.56) resulted in a z-score of 0.63, land use and forestry (*r* = 0.61) resulted in a z-score of 0.70, agricultural activities (*r* = 0.54) resulted in a z-score of 0.60, waste (*r* = 0.60) resulted in a z-score of 0.69, other energy production (*r* = 0.64) resulted in a z-score of 0.75, sports organizations (*r* = 0.57) resulted in a z-score of 0.64, and carbon footprint awareness scale (*r* = 0.81) resulted in a z-score of 1.12. Electricity and heat production, land use, and forestry (*r* = 0.61) resulted in a z-score of 0.70, agricultural activities (*r* = 0.54) resulted in a z-score of 0.60, waste (*r* = 0.60) resulted in a z-score of 0.69, other energy production (*r* = 0.64) resulted in a z-score of 0.75, sports organizations (*r* = 0.57) resulted in a z-score of 0.64, and carbon footprint awareness scale (*r* = 0.81) resulted in a z-score of 1.12. Land use and forestry and agricultural activities (*r* = 0.40) resulted in a z-score of 0.42, waste (*r* = 0.52) with a z-score of 0.57, other energy production (*r* = 0.57) with a z-score of 0.64, sports organizations (*r* = 0.47) with a z-score of 0.51, and carbon footprint awareness scale (*r* = 0.74) with a z-score of 0.95. Agricultural activities and waste (*r* = 0.51) resulted in a z-score of 0.56, other energy production (*r* = 0.48) with a z-score of 0.52, sports organizations (*r* = 0.60) with a z-score of 0.69, and carbon footprint awareness scale (*r* = 0.73) with a z-score of 0.92. Waste, with other energy production (*r* = 0.50), resulted in a z-score of 0.54, sports organizations (*r* = 0.50), resulted in a z-score of 0.54, and the carbon footprint awareness scale (*r* = 0.74) resulted in a z-score of 0.95. Other energy production, with sports organizations (*r* = 0.56), resulted in a z-score of 0.63, and carbon footprint awareness scale (*r* = 0.78) resulted in a z-score of 1.04. Sports organizations with a carbon footprint awareness scale (*r* = 0.80) resulted in a z-score of 1.09 (Table 8).

## Conclusions

In this study, a measurement tool was developed to measure carbon footprint awareness among university students validly and reliably. In order to develop the measurement tool, a literature review was conducted, expert opinions were received, and a pool of 146 items was created. A 50-item draft scale was created in line with the item pool, pilot application, and expert opinions. Exploratory Factor Analysis (EFA) and Confirmatory Factor Analysis (CFA) were applied in order to ensure the construct validity of the Carbon Footprint Awareness Scale. In order to determine the suitability of the data for factor analysis, the calculated KMO measurement value was 0.948, and the Bartlett Sphericity test chi-square value was χ2 = 15474.121 (df = 1431, *p* < 0.000). Kaiser-Meyer-Olkin is a criterion for the factorability of the variables (items) subjected to analysis^76^. Bartlett’s Sphericity Test includes testing whether the difference between the inter-item correlation matrix and the unit matrix is significant to accept that the data is suitable for analysis^45^. KMO values above 0.60 are acceptable values. Bartlett’s test should be statistically significant (*p* < 0.05). Tavşancıl^77^ states that if the value found due to the KMO test is below 0.50, analysis cannot be performed, and values of 0.90 and above are perfect. Similarly, studies indicate that a KMO value between 0.90 and 1.00 indicates perfect favorability^45,77^. In this context, it can be said that the KMO value found in the study indicates that the scale is perfect for factor analysis. Principal Components Analysis and the varimax rotation technique were used while performing factor analysis. In the EFA (Exploratory Factor Analysis) stage, the “principal components analysis” method and Sree Plot for EFA were used. The lower cut-off point in the rotation technique was taken as 40. In this context, it was seen in the EFA that the draft scale consisted of ten sub-dimensions. Following the literature, the sub-dimensions of the carbon footprint awareness scale were named building, fossil fuel transportation, transportation, petroleum refining, industry, electricity, heat production, land use and forestry, agricultural activities, waste, other energy, and sports organization. The building sub-dimension consisted of items 1, 2, 3, 4, and 5. This subdimension explained 10% of the variance, and the Cronbach’s alpha value was 0.783. Fossil fuel transportation and transportation sub-dimension comprised items 6, 7, 8, 9, 10, 11, and 12. This subdimension explained 18% of the variance, and the Cronbach’s alpha value was 0.873. The petroleum refining subdivision comprised items 13, 14, 15, and 16. This subdimension explained 26% of the variance, and the Cronbach’s alpha value was 0.857. The industry subdimension comprised items 17, 18, 19, 20, and 21. This subdimension explained 32% of the variance, and the Cronbach’s alpha value was 0.826. The electricity and heat production subdimension consisted of items 22, 23, and 24. This subdimension explains 39% of the variance, and the Cronbach’s alpha value is 0.807. The land use and forestry subdimension consists of items 25, 26, 27, and 28. This subdimension explains 44% of the variance, and the Cronbach’s alpha value was determined as 0.790. The agricultural activities subdimension consists of items 29, 30, 31, 32, 33, 34, and 35. Waste subdimension consists of items 36, 37, 38, and 39. This subdimension explains 55% of the variance, and the Cronbach’s alpha value is 0.781. The other energy subdimension consists of items 40, 41, 42, and 43. This sub-dimension explains 59% of the variance, and the Cronbach’s alpha value is 0.864. The sports organization sub-dimension consists of items 44, 45, 46, 47, 48, 49, and 50.This sub-dimension explains 62% of the variance, and Cronbach’s alpha value is 0.910. As a result of EFA, 50 items explaining 62.245% of the total variance were obtained. It is sufficient for the explained variance rate to be between 40% and 60%. It can be said that the variance rate obtained in the study is quite good. The factor loading values of the items in the measurement tool vary between 0.43 and 0.72. Harrington^78^ states that factor loadings should not be below 0.30, 0.32-weak, 0.45-good/acceptable, 0.55-good, 0.63-very good, and 0.71 and above should be evaluated as excellent. It can be said that the factor loadings of the items obtained in the research provide evidence that they are good at representing the dimensions in which the items are located. In the confirmatory factor analysis results of the Carbon Footprint Awareness Scale, the fit index values related to the Carbon Footprint Scale were determined as χ2/df = 2.79, the NFI value is 0.96; the NNFI value is 0.97; the GFI value is 0.91, the CFI value is 0.97, and the AGFI value is 0.86. According to the acceptable variance range and the good variance range, the 10 sub-dimensions obtained from the confirmatory factor analysis appear to have adequate fit indices^60,68–70,72,79^. A modification process was carried out for the scale developed in the study. The analysis showed that the fit indexes were between good and acceptable fit values^68^. One (1) modification process was carried out.

When the correlation analysis of the carbon footprint awareness scale sub-dimension scores is examined, the correlation coefficients resulting from the analysis are a value that measures the degree of relationship between the dimensions and vary between − 1 and + 1. When these values are close to -1, they show a powerful negative and linear relationship between the scale dimensions; when they are close to -1, they show a powerful and positive linear relationship^80^. When Table 8 is examined based on this information, it is determined that a high level of relationship between the sub-dimensions and internal consistency is achieved.

To test the scale’s reliability, the internal consistency coefficient of the scale was calculated, and it was stated that the scales with an internal consistency coefficient between 0.70 and 1 in the overall scale have high reliability^33,42,61^. In this context, based on the reliability analysis of the current scale, it was proven that the overall scale and its sub-dimensions were highly reliable. As a result, it was determined that the Carbon Footprint Awareness Scale was a valid and reliable measurement tool.

As a result, Rapid population growth, industrialization, agriculture, and urbanization have begun to strain the carrying capacity of the natural environment, non-renewable resources have been rapidly depleted, renewable resources have been destroyed, and negative problems concerning the future of man have increased. If there is no change in the usual production and consumption habits, there will be no world where the next generations can continue their lives. For this reason, understanding that natural resources are not unlimited and preferring environmentally friendly technologies and behaviors instead of current production and consumption habits has become necessary^81,82^. The first step in reducing the effects on natural systems is awareness: Accepting the existence of environmental problems, clarifying what the sources of the problem are, searching for solutions in this direction, and putting them into practice. In order to increase the awareness mentioned and create behavioral changes, it is essential to measure man’s direct and indirect effects on nature. Carbon footprint awareness is of great importance at this point. With the current study, carbon footprint awareness will contribute to forming social consciousness among university students. This will increase awareness about the source of carbon emissions and taking precautions. With the carbon footprint awareness scale, consumption behaviors will be more conscious, and people will be informed about the environmental damage the products they consume cause. Large amounts of energy are used in raw materials from industry to textiles. Thanks to recycling, the need for raw materials will decrease, and energy consumption will decrease. This will help protect natural resources and contribute to sustainability.

Nowadays, it is important to measure carbon footprints and determine individuals’ tendencies and awareness to draw attention to increasing environmental problems and to ensure that individuals’ effects on the formation of environmental problems are recognized. The current study will contribute to the field and strengthen the literature by completing the missing parts of previously developed scales. For example, in the relevant literature, the Ecological Footprint Awareness Scale was developed by Coşkun and Sarıkaya^82^. Has five sub-dimensions, 40 items, and a 5-point Likert-type scale, including food, transportation-shelter, energy, waste, and water consumption. The current study’s carbon footprint awareness scale has 10 sub-dimensions, 50 items, and a 7-point Likert-type scale. It will allow the examination of subjects with more different sub-dimensions than other scales.

### Suggestions

- According to our results, the sample group (company employees, sports business employees, athletes) can be changed, and how carbon footprint awareness levels change over 5 years with the training and seminars provided can be monitored.
- Education policies can be organized and provided in schools, especially for university students, to reduce the amount of carbon dioxide in the air.
- Education courses on environmental pollution, climate change, and carbon footprint can be organized in line with state policies on carbon footprint and environmental pollution.

### Limitations of the study

Only Turkish university students were included in the study.

Individuals over the age of 18 were included in the study.

Only the personal information form and carbon footprint awareness scale were used in the study.

While university students represent an accessible and often-used population in scale development, we agree that future research should include more diverse samples (e.g., general population, different cultures) to enhance generalizability and test for potential sample bias.

In this study, construct validity was addressed only in the first phase of the scale development process, and the scale’s factor structure was tested through exploratory and confirmatory factor analyses (EFA and CFA). However, other types of validity, such as criterion-related and convergent, were not assessed. The primary reason for this limitation is the absence of a widely accepted external criterion in the literature related to carbon footprint awareness, which restricts the possibility of conducting criterion-related validity analyses. Therefore, it is recommended that future studies examine these types of validity using different samples and comparative measurement tools.

### Recommendation on future research

As a result of the study, the Carbon Footprint Awareness Scale was developed as a reliable and valid instrument, demonstrating strong construct validity and high internal consistency (Cronbach’s Alpha = 0.966). The scale offers a multidimensional structure with ten distinct factors and can be used to assess university students’ awareness regarding the sources and impacts of their carbon footprint.

However, some aspects of the validation process remain open for future exploration.

First, criterion-related and convergent validity analyses were not included in this initial development phase due to the absence of a universally accepted external measurement tool in the literature. Future research should focus on validating the scale against related instruments or observable behavioral indicators to strengthen external validity.

Second, the current study used a sample limited to university students. Replicating the study with diverse populations, including high school students, working adults, and individuals from different cultural or geographical backgrounds, would help assess the scale’s generalizability across different demographic groups.

Third, longitudinal studies could be designed to test the scale’s sensitivity to change over time and its ability to measure the effectiveness of educational or environmental intervention programs to raise carbon footprint awareness.

Fourth, future studies might examine the relationship between scores on this scale and actual environmental behaviors (e.g., energy consumption, transportation choices, recycling habits) to determine the scale’s predictive validity.

Fifth, qualitative research methods (e.g., interviews, focus groups) could be used in future work to gain deeper insights into the dimensions identified in the scale and explore potential new dimensions that may not have emerged in this study.

Finally, integrating the scale into educational curricula or awareness campaigns and measuring its practical impact on awareness levels could enhance its utility in real-world applications and provide further evidence of its effectiveness.

## Data Availability

The original contributions presented in this study are included in the article. Further inquiries can be directed to the corresponding author.

## Abbreviations

CFAS: Carbon Footprint Awareness Scale
KMO: Kaiser-Meyer Olkin
EFA, CFA: Exploratory and confirmatory factor analysis
